# Orthodontic Treatment Characteristics and Outcomes in an Educational Setting

**DOI:** 10.1155/2020/8367232

**Published:** 2020-04-30

**Authors:** T. Al-Jewair, V. Ryan, S. Warunek

**Affiliations:** ^1^Department of Orthodontics, School of Dental Medicine, State University of New York, Buffalo, NY, USA; ^2^Private Practice, LaVale, MD, USA

## Abstract

**Background:**

To assess and correlate orthodontic treatment characteristics and outcomes in an educational setting.

**Methods:**

A total of 287 patients were included. Independent chart reviews were conducted to gather demographic and pretreatment diagnostic information. Posttreatment digital records were graded with the ABO C-R Eval and the CCA methods. Pearson correlation coefficients were calculated to determine associations between variables.

**Results:**

Of the 287 patients, 122 (42.5%) were male and 165 (57.5%) were female. The total average treatment time was 33.87 ± 10.28 months, with a range from 11 to 75 months. The mean ABO C-R Eval score was 29.10 ± 8.59 points. The parameters with the highest scores were buccolingual inclination and occlusal contacts. The mean CCA score was 3.36 ± 2.05 points. The highest scores were recorded for dental esthetics and management of the periodontium. Higher ABO DI scores were weakly correlated with longer treatment times (*r* = 0.258; *p* < 0.001). ABO C-R Eval scores showed a weakly significant association with treatment duration (*r* = 0.162; *p*=0.006), while CCA scores were moderately associated with treatment duration (*r* = 0.451; *p* < 0.001).

**Conclusions:**

As treatment duration increased, the total ABO C-R Eval and CCA scores tended to increase; thus, quality of treatment outcomes decreased. A significant positive correlation was also found with the ABO DI score and treatment duration.

## 1. Background

Several indices have been utilized to objectively assess clinical outcomes of orthodontic treatment such as the American Board of Orthodontics Cast-Radiograph Evaluation (ABO C-R Eval), the Peer Assessment Rating (PAR) index, and the Comprehensive Clinical Assessment (CCA) [1–9]. The ABO C-R Eval and the PAR index do not take into account resulting facial esthetics, duration of treatment, or iatrogenic changes such as enamel scarring [1, 3, 5]. Therefore, the CCA was developed in 2004 by the Indiana University School of Dentistry (IUSD) orthodontics program to address these limitations [4]. The CCA measures seven posttreatment variables: facial esthetics, dental esthetics, vertical control, arch-form, periodontal management, root structure preservation, and treatment efficiency. Multiple studies have included the CCA method in their assessment of treatment outcomes since its development [2, 4, 5, 10]

There have been several studies done at advanced education orthodontics programs to evaluate treatment outcomes over time in their clinics [2, 4–8, 10–12]. Pinksaya et al. [4] assessed treatment outcomes using the ABO C-R Eval and CCA on 521 posttreatment records. They determined that, as treatment duration increased in their clinic, the quality of treatment outcomes decreased, which they attributed to “patient burn-out” and lack of cooperation and compliance [4]. Two subsequent studies [3, 6] were published assessing treatment outcomes at later times in the same clinic and after implementing improvements [2]. They all reported improvements in treatment outcomes since the first baseline study. They also determined that the average passing rate of these cases for the ABO clinical examination increased significantly from only 39.7% (1998–2000) to 76.6% (2001–2003) and 55.6% (2004–2006). Deguchi et al. [10] from Okayama University Department of Orthodontics in Japan determined that 45% of the finished patients would likely pass the ABO exam. Palmer [12] at Nova Southeastern Clinic reported a 34% passing rate. Their results also showed that a higher DI score is associated with a longer treatment time but not necessarily a poorer result.

With the advances in orthodontic technology, one would expect an improvement in treatment outcomes. Yet, there is paucity of recent studies that assess treatment outcomes in an educational setting. The aims of this study were to evaluate orthodontic treatment characteristics and outcomes in an advanced orthodontic program. We hypothesized that patients with higher ABO DI index scores will have longer duration of treatment, and patients with longer treatment durations will have poorer treatment outcomes.

## 2. Materials and Methods

This is a retrospective chart review of patients that completed orthodontic treatment at the UBSDM orthodontics advanced education program. Approval was obtained from the University at Buffalo Institutional Review Board (#00000740).

Orthodontic patients of all ages, genders, and ethnicities who completed orthodontic treatment between June 2013 and 2016 were screened for inclusion. Only patients who had complete pre- and posttreatment records of adequate quality, namely, digital models, intra- and extraoral photographs, panoramic radiographs, and lateral cephalograms, were included. All treatment categories were considered including interceptive, surgical, and craniofacial orthodontic treatments. Patients were excluded from the sample if they were in treatment or if their records were of inadequate quality.

One investigator (V.R.) collected and measured data from patient charts, their digital records on the Dolphin Imaging software version 11.7 and their digital models on the OrthoCAD software version 5.4.

Pretreatment data included demographic and orthodontic diagnostic information such as type of malocclusion, missing teeth and types, impacted teeth, treatment phase (one-phase or two-phase), and ABO DI score. Treatment and posttreatment variables included the ABO C-R Eval, CCA, treatment duration in months, extraction treatment, early debond, year patient was debonded, type of orthodontic appliance, type of functional appliance used, number of missed appointments, appliance maintenance, oral hygiene, and whether any multidisciplinary care was administered.

Posttreatment digital models were measured to assess the ABO C-R Eval scores using the ABO Phase III grading tool on OrthoCAD software version 5.4. Dolphin Imaging software was used to evaluate the root alignment on the posttreatment panoramic radiographs.

The CCA measurements were conducted using the Dolphin Imaging software. Initial photos were compared with final photos to determine any deductions for facial and dental esthetics, vertical control, and periodontium management. Pretreatment panoramic radiographs were compared with posttreatment ones to score any points for bone loss or root structure preservation. Root resorption was measured using the Malmgren Root Resorption Index^42^. Moderate root resorption was scored if 2 mm or 1/3 of the root was resorbed, and greater than 1/3 of the root was considered severe root resorption [13]. The treatment duration was obtained from patient's charts. Points were deducted for any case with treatment duration of greater than 36 months. This time point was selected as the baseline because it is the length of the residency program, and the overall goal is for residents to be able to finish their started cases within these 36 months of residency, minus any extenuating circumstances.

### 2.1. Intraexaminer Reliability

The investigator was calibrated for the ABO C-R Eval by a hands-on, three-hour session with an ABO examiner, and the information was provided in the OrthoCAD version 5.4 software package on the ABO phase III grading tool. The CCA scores and the ABO C-R Eval scores for 10 randomly selected cases were measured and repeated 3 weeks later to assess intraexaminer reliability. The Intraclass correlation coefficients (ICC) indicated that the measurements were reproducible (Table 1).

### 2.2. Pilot Study

The validity and reliability of measuring the ABO C-R Eval scores with the OrthoCAD software have been assessed in previous studies [14, 15]. Previous studies however were conducted using older versions of the software. A pilot study was therefore conducted to determine the reliability of using the OrthoCAD Version 5.4 software for the ABO C-R Eval grading prior to outcome assessment. 10 posttreatment models of good finish were randomly selected and printed to ABO specifications with a digital printer at an orthodontic lab. Manual and digital (using the ABO ruler and the ABO Phase III grading tool on the OrthoCAD Version 5.4 software) measurements were then conducted and compared. The reproducibility was shown to be high (ICC = 0.981 (95% CI 0.927–0.995); *p* < 0.001).

### 2.3. Statistical Analysis

Data were analyzed using IBM SPSS v. 21 for Windows. Descriptive statistics were conducted, and the ABO C-R Eval and CCA scores were reported in means with standard deviations and percentages. The passing score for the ABO clinical exam used to be ≤27 points, and this was the cutoff point used to determine a passing or failing score for each case. Independent sample *t*-tests were used to determine differences between treatment outcomes and different variables such as extraction, early debond, and treatment duration. Analysis of variance (ANOVA) with post hoc Tukey's-b tests was used to assess differences in treatment outcomes between the individual years studied. Pearson correlation coefficients (*r*) were calculated to assess the relation between treatment duration, treatment outcomes, and ABO DI scores. The significance level was set at 5% using a two-tailed test.

## 3. Results

From a sample of 633 patients debonded, a total of 287 complete patient records were included. Of these, 122 (42.5%) were male and 165 (57.5%) were female. The males on average were nearly 1 year older than females (mean ± SD = 15.89 ± 6.94 yrs vs. 15.11 ± 5.78 yrs) (Table 2). However, this difference was not statistically significant, *p*=0.249.

29 (10.1%) patients had missing teeth, with the mandibular second premolars most commonly missing (28%). 35 (12.2%) patients had impacted teeth. The most commonly impacted tooth was the upper right (15; 42.8%) followed by the upper left cuspid (11; 31.4%). 25 patients (8.7%) received Phase I followed by Phase II treatment with a period of retention in between. Of the Phase II treatment patients, 255 (95.3%) received fixed orthodontic appliances with 0.018” bracket slot, MBT prescription. 52 (19.8%) received functional appliances including Herbst, MARA, Twin-block, and Headgear. 60 (20.9%) cases received multidisciplinary care such as cosmetic bondings or canine exposure procedures and surgical and craniofacial treatment. 54 (18.8%) patients terminated treatment early due to lack of compliance with attendance, appliance maintenance, oral hygiene, or at patient or parental/guardian's request. The mean number of missed appointments was 6.6 ± 4.88.

The total average treatment time was 33.87 ± 10.28 months, with a range from 11 to 75 months (Table 3). 63.1% of patients in the sample finished their treatment in just under 36 months. Patients who were treated with extractions had, on average, 6 months' longer treatment than those who were not (38.56 ± 10.12 months vs. 32.26 ± 9.85 months); however, this difference was not statistically significant (*p*=0.638), Table 4.

**Figure 1:**
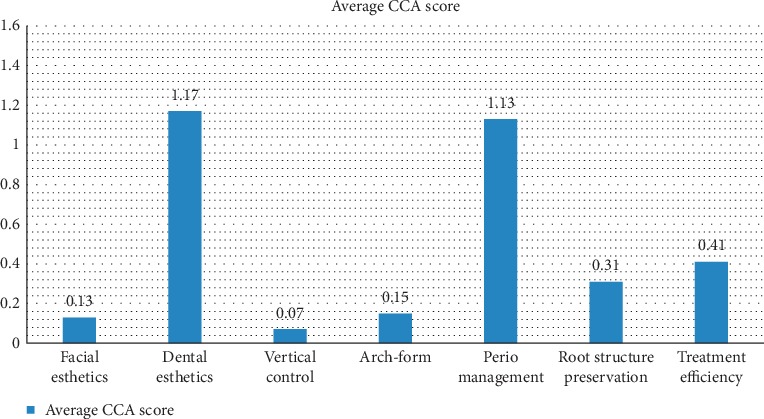
Average CCA score per component.

The average CCA score for the entire sample was 3.36 ± 2.05, with a range from 0 to 15 points.  Figure 1 shows the mean for each of the seven components of the CCA system. The parameter that received the highest score (worst performance) was dental esthetics, followed closely by management of the periodontium.

In looking at the scores for dental esthetics, 157 (54.7%) patients showed signs of enamel scarring or residual bonding resin after treatment, and 17 (6%) patients showed active decalcifications after debonding. 129 (45%) patients had points deducted for deviations from ideal in dentition at embrasures, contours, incisal edges, black triangles, or buccal corridors. For periodontium management, 87 (30.3%) patients had signs of moderate gingivitis and 43 (15%) severe gingivitis posttreatment. 82 (28.6%) patients showed moderate recession, and 27 (9.4%) had severe recession. The scores for treatment efficiency showed that 60 patients (20.9%) exceeded expected treatment time—28 patients exceeded expected treatment time by 6 months, 21 exceeded by 12 months, and 11 exceeded by 18 months. However, only 11 patients (3.8%) showed a moderate or severe compromise in the treatment result relative to treatment time. For root structure preservation, the highest occurrence was in the incisor region—58 patients (20.2%) showed moderate resorption of incisors, and 11 patients (3.8%) showed severe incisal resorption posttreatment. 36 patients (12.5%) scored points under “arch-form” for dentition not centered over the basilar bone and apical base. The facial esthetics and vertical control both received fewest points.

**Figure 2:**
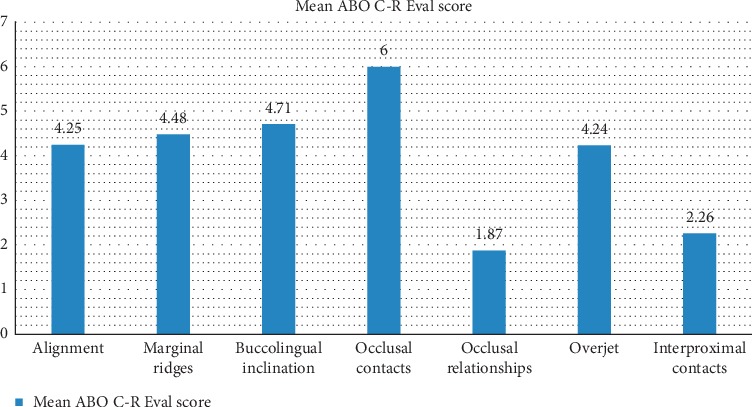
Average ABO OGS score per cast parameter.

The average ABO C-R Eval score for the entire sample was 29.10 ± 8.59 points, and 130 patients (45.3% of cases) received a score of 27 or less. The cast parameter that had the highest score was occlusal contacts (6.00 ± 3.59), followed by buccolingual inclination (4.71 ± 2.71), while the lowest scoring parameter was occlusal relationships (1.87 ± 1.81), Figure 2.

When the treatment outcomes were compared against early treatment termination, the results showed a statistically significant difference in CCA scores for patients whose treatment was terminated early versus patients whose treatment was not (*p* < 0.001), indicating that they had poorer performance than those who were treated to completion, Table 5. Also, there was a statistically significant difference in the average CCA score among the 4 years (*p* < 0.001). Post hoc testing determined that year 2013 had significantly lower mean CCA scores than the other 3 years studied (1.74 ± 1.44 points). Year 2015 tended to have the highest mean CCA score at 3.86 ± 2.38 points.

We found a statistically significant positive correlation between the ABO DI score and treatment duration (*r* = 0.258; *p* < 0.001), Table 6. Although significant, the strength of this correlation was weak. There was also a statistically significant positive correlation for both ABO C-R scores (*r* = 0.162; *p* < 0.05) and CCA scores (*r* = 0.451; *p* < 0.001) and treatment duration. Patients with longer treatment times had poorer treatment outcomes. As a result, both hypotheses were not rejected.

## 4. Discussion

This study assessed treatment characteristics and outcomes in an advanced education orthodontics clinic. The mean ABO C-R Eval score was 29.10 points, which is consistent with the published data from University of Detroit Mercy (28.38 points) [7] but slightly lower than most of the other advanced education orthodontic programs [4–6, 10, 12]. It should be noted that the passing criteria for ABO C-R Eval are varied among studies ranging from <20, <27 to <30 which could affect the passing percentage.

Based on the ABO's cutoff point of 27 for finishing with good quality and previously passing the ABO phase III examination, 130 (45.3%) cases were treated to “board quality”. Affecting this percentage are the 54 patients in our sample who had their treatment terminated early and were thus not finished to ideal standards. The average ABO C-R Eval score for the “early debond” patients was significantly higher at 33.04 points, so these cases likely increased the average score for the entire sample. When the 54 “early debond” patients are removed from the sample, the average ABO C-R Eval score was lowered to 28.19 points.

When looking at the individual components of the ABO C-R Eval system, the patients had the highest scores in occlusal contacts and buccolingual inclination, which are similar to findings at other universities [2, 4–6]. It is standard practice in our clinic to take an intraoral scan for final models the same day the appliances are removed. However, delaying final scan to the first retention check (about 1 month after braces are removed) may improve the occlusal contact scores, but this could also pose a risk of loss of complete records for future studies if patients do not return.

Another high-scoring component showing poor results was buccolingual inclination. Yang-Powers et al. [6] also found high deductions in buccolingual inclination with an increased use of the preadjusted bracket prescription in their orthodontics program. The main reason for higher scores in buccolingual inclination in our sample was improper inclination of second permanent molars. In fact, this caused the majority of deductions in not only buccolingual inclination but also occlusal contacts, marginal ridges, and alignment. There are several reasons why second molars pose an issue. Some patients may begin treatment before their second molars are fully erupted into the arch, so these may not be engaged until later in treatment, and it is not always reasonable to extend the treatment time until they are ideally positioned. Also, if the second molars are fully erupted and seem to be in a good position, including them in treatment may not seem necessary, and thus, their position may not change.

The mean CCA score in our sample was 3.36 points, which is slightly lower than the previously published studies at IUSD: 4.67 points; [4] 4.38 points; [2] and 5.62 points [6] but more consistent with the average scores from Okayama University in Japan (3.9 points) [10]. The 54 “early debond” patients had a significantly higher CCA score of 4.83 points when compared with the patients with typical treatment times. The CCA method is driven by esthetics, so including patients who were not treated to ideal outcomes is likely to raise the average score for the whole sample. When these were removed from the analysis, the average CCA score dropped to only 3.02 points. It is also important to note that, although 2013 showed, on average, lower CCA scores indicating better treatment outcomes, the sample in this group was small (19 patients; 6.6%) due to high record exclusion for different reasons, and the smaller number of patient starts as compared to subsequent years.

The parameters of the CCA that received the highest scores and poorest outcomes in this study were dental esthetics and management of the periodontium. Dental esthetics is an important aspect of patients seeking orthodontic treatment. Nearly half of the patients (45%) had deviations from ideal microesthetics (uneven incisal edges, gingival margins, or embrasures; black triangles or excessive buccal corridors). The most common dental esthetics problem was enamel scarring (55%). Dental esthetics also posed the biggest challenge to the orthodontics programs at IUSD and Okayama University [2, 4, 5, 10]. Even the follow-up study done at IUSD after the implementation of changes in clinical protocol still showed this parameter was the most difficult for residents to achieve successful outcomes [2]. Pinksaya et al. [4] highlights the importance of “esthetic detailing” during treatment such as selective smoothing and grinding of incisal edges, rounding of incisal embrasures, and referring for additional restorations as needed if esthetics are poor and beyond the help of the orthodontist.

Periodontal management was the second-highest scoring component of the CCA total score, with 45% of patients showing moderate-to-severe gingivitis at the end of treatment. Based on the results of our study, a greater emphasis was placed in the clinic on giving oral hygiene instructions at every patient visit. The lowest components of the CCA score were vertical control and facial esthetics, which were consistent with published studies [2, 4, 5].

The average duration of treatment from 2013 to 2016 was 33.8 months, just under the expected treatment time of 36 months. Although the difference in mean treatment times between groups was not statistically significant, patients who had extractions during treatment tended to have, on average, a 6-month increase in treatment duration. This is consistent with findings in Pinksaya et al. [4] (4-month increase) and Knierim et al. [2] (4.78-month increase) with regard to extraction treatment. When removing these extraction patients with longer treatment times from the sample, the average treatment duration drops down to 32.3 months.

Our study found a statistically significant correlation between treatment outcomes and treatment duration. As the treatment times increased, the ABO C-R Eval and CCA scores increased. The correlation however was weak and might have been confounded by case complexity, reflected by the ABO DI scores. The mean ABO C-R Eval score for patients with a treatment duration of more than 36 months was 1 point higher than those treated in the optimal treatment time, and the CCA score was increased by almost 2 points in this group, which is understandable because this method measures overall treatment result relative to treatment time, and prior studies have shown that it is more sensitive than the ABO C-R Eval for detecting deteriorating treatment outcomes with excessive treatment times [2, 11]. While most residents would like to ideally finish their patients to completion, if we note problems with compliance and are facing the decision whether to transfer these patients to new residents and continue on with treatment, it is likely in the patient's best interest to terminate treatment early. By extending their treatment times, they are more likely to experience issues with oral hygiene, enamel scarring, decalcifications, and poor gingival health, which were shown in this sample by higher scores in these categories and overall CCA score when treatment times were increased.

In agreement with Parrish et al.'s study [16], our study found a significant correlation between ABO DI scores and treatment duration. Although significant, the correlation was weak, which could be attributed to the limitations in the ABO DI in terms of underscoring cases with craniofacial anomalies and impacted canines. Parrish et al. [16] found that, for every point increase in the total DI score, the treatment time was lengthened by about 11 days. Thus, a 10-point increase in the DI score would translate to an additional 110 days of treatment.

Table 7 presents the mean treatment quality and efficiency across advanced education orthodontic programs. The mean ABO C-R Eval and treatment duration ranged from 25.1 ± 11.9 to 45.5 ± 18.3 and 31.7 ± 15.7 to 42 ± 15, respectively. The most common area of improvement across orthodontic programs was the alignment of the maxillary and mandibular second molars. Some of the reasons explained were the second molars' anatomical position that makes it difficult to properly position or move in addition to late banding of these teeth [13]. Campbell et al. [7] added that “lack of occlusal contacts and poor treatment efficiency” lead to deficiencies in clinical outcomes in their program. Additionally, Deguchi et al. [10] believe that patients should be happy and satisfied with their smile through orthodontic treatment and consequently suggested measuring patient expectations and levels of satisfaction throughout treatment. Abei et al. explained that satisfaction can be influences by multiple factors including the provider and staff personalities, as well as the office appearance [17].

Although case submission to the ABO for Phase III clinical examination is no longer a requirement, the development of a common clinical protocol for programmatic outcome monitoring and evaluation across advanced education programs are recommended.

Based on this study and previous studies in university settings on the implementation of clinical protocols to improve treatment outcomes, [2, 7, 12] the following changes were proposed: assignment of patients based on ABO DI scores, routine prefinish records and review with overseeing faculty, bonding of second molars early in treatment, reviewing the debonding protocol, complete chart documentation and record taking, and more frequent review of residents' patient families to increase treatment efficiency.

## 5. Conclusions

The average ABO C-R Eval and CCA score for the sample were 29.10 ± 8.59 and 3.36 ± 2.05 points, respectively. 63% of patients finished treatment within the expected time of 36 months. As treatment duration increased, the total ABO C-R Eval and CCA scores tended to increase; thus, quality of treatment outcomes decreased. A significant positive correlation was also found with the ABO DI score and treatment duration.

## Figures and Tables

**Table 1:** Intraexaminer reliability.

		95% confidence interval		
Treatment outcome	ICC	Lower bound	Upper bound	Sig.
CCA scores	0.978	0.913	0.994	<0.001
ABO C-R Eval scores	0.977	0.911	0.994	<0.001

^*∗*^ICC = intraclass correlation coefficient.

**Table 2:** Sample characteristics (*N* = 287).

Variable		*N*	Percent
Age	18 and under: ≤18 yrs	244	85.0
	older than 18: >18 yrs	43	15.0

Sex	Male	122	42.5
	Female	165	57.5

Ethnicity	White	238	82.9
	Hispanic	7	2.4
	Asian	9	3.1
	Black	30	10.5
	Other	3	1.0

ABO DI score	<10	79	27.5
	10 to 20	134	46.7
	>20	74	25.8

Malocclusion	Class I	129	44.9
	Class II div 1	93	32.4
	Class II div 2	42	14.6
	Class III	23	8.0

Missing teeth	No	258	89.9
	Yes	29	10.1

Impacted teeth	No	252	87.8
	Yes	35	12.2

Extraction treatment	No	214	74.6
	Yes	73	25.4

Interceptive phase	No	262	91.3
	Yes	25	8.7

Year debonded	2013	19	6.6
	2014	110	38.3
	2015	93	32.4
	2016	65	22.6

Early debond	No	233	81.2
	Yes	54	18.8

Treatment duration	Less than 36 m	181	63.1
	More than 36 m	106	36.9

**Table 3:** Mean treatment duration, ABO DI scores, and treatment outcomes.

Variable	*N*	Mean	Std. dev.	Min.	Max.
Treatment duration (months)	287	33.87	10.28	11.21	75.26
DI score	287	15.67	9.08	0.00	51.00
CCA score	287	3.36	2.05		
ABO C-R Eval	287	29.10	8.59		

“*Board quality*” *scores*					
ABO C-R < 27 points	130	22.13	4.22	10.00	27.00
ABO C-R ≥ 27 points	157	34.88	6.83	28.00	72.00

*ABO DI score*					
Class I	129	11.75	7.45	2.00	49.00
Class II div 1	93	19.28	8.97	4.00	46.00
Class II div 2	42	17.52	7.62	4.00	33.00
Class III	23	19.70	11.61	0.00	51.00

**Table 4:** Treatment duration with extraction vs. nonextraction treatments.

	Extraction	*N*	Mean ± SD	Sig.
Treatment Duration (months)	Yes	73	38.6 ± 10.1	0.638
	No	214	32.3 ± 9.9	

**Table 5:** Bivariate analysis of treatment outcomes and categorical variables.

		ABO C-R Eval		CCA	
Variable	*N*	Mean ± SD	Sig.	Mean ± SD	Sig.
*Year*			0.060		<0.001
2013	19	28.2 ± 8.5		1.7 ± 1.5	
2014	110	27.9 ± 8.4		2.9 ± 1.5	
2015	93	31.1 ± 9.3		3.9 ± 2.4	
2016	65	28.5 ± 7.7		3.8 ± 2.1	

*Early debond*			<0.001		<0.001
No	233	28.2 ± 7.6		3.0 ± 1.9	
Yes	54	33 ± 11.3		4.8 ± 2.2	

*Treatment Duration*			0.187		<0.001
<36 months	181	28.6 ± 8.7		2.9 ± 1.5	
≥36 months	106	29.9 ± 8.5		4.2 ± 2.5	

*Age*			0.089		0.498
≤18 yrs	244	29.5 ± 8.3		3.3 ± 2.1	
>18 yrs	43	27.1 ± 9.9		3.6 ± 1.7	

**Table 6:** Pearson correlation coefficients (*r*) between ABO DI, treatment outcomes, and treatment duration in months.

	Treatment duration (months)	
	*r*	Sig.^*∗*^
ABO DI score	0.258	<0.001
ABO C-R Eval score	0.162	0.006
CCA score	0.451	<0.001

^*∗*^ *P* < 0.05.

**Table 7:** Mean treatment quality and efficiency across advanced education orthodontic programs (by publication date).

		ABO C-R Eval	CCA	Tx duration (mon)	ABO DI score
*Authors*	*N*	Mean ± SD	Mean ± SD		
Yang-powers et al., 2002	92	45.5 ± 18.3	—	35.1 ± 10.5	—
Pinksaya et al., 2004	521	34.4 ± 10.4	4.7 ± 3.0	33.9 ± 14.1	—
Hsieh et al., 2005	408	34.8 ± 10.5	2.7 ± 2.3	—	—
Deguchi et al., 2005	72	33.6 ± 13.6	3.9 ± 2.5	34.6 ± 10.4	19.1 ± 12.9
Cook et al., 2005	77	25.1 ± 11.9	—	31.8 ± 9.6	—
Knierim et al., 2006	437	25.2 ± 11.2	4.4 ± 2.7	36.2 ± 16.1	—
Campbell et al., 2007	382	32.6 ± 13.9	5.6 ± 2.7	42 ± 15	20.9 ± 12.2
Brown et al., 2011	714	28.4 ± 5.0	—	—	15.5 ± 3.1
Palmer, 2012	295	33.9 ± 12.2	—	31.7 ± 15.7	18.3 ± 10.3
Current study, 2018	287	29.1 ± 8.6	3.4 ± 2.1	33.9 ± 10.3	15.7 ± 9.1

## Data Availability

All data generated or analyzed are curated by the principal investigator.

## Abbreviations

ABO C-R Eval: American Board of Orthodontics Cast-Radiographic Evaluation
CCA: Comprehensive clinical assessment
IUSD: Indiana University School of Dentistry
ABO DI: American Board of Orthodontics Discrepancy Index
PAR: Peer Assessment Rating
ICC: Intraclass correlation coefficients
ANOVA: Analysis of variance.
